# An adaptive response to uncertainty can lead to weight gain during dieting attempts

**DOI:** 10.1093/emph/eow031

**Published:** 2016-12-05

**Authors:** A. D. Higginson, J. M. McNamara

**Affiliations:** 1 Centre for Research in Animal Behaviour, College of Life and Environmental Sciences, University of Exeter, Exeter EX4 4QG, UK; 2 Previous address: School of Biological Sciences, Life Sciences Building, University of Bristol, 24 Tyndall Avenue, Bristol BS8 1TQ, UK; 3 School of Mathematics, University of Bristol, University Walk, Bristol BS8 1TW, UK

**Keywords:** obesity, optimal foraging, contrast effect, low calorie diets, yo-yo dieting, weight cycling

## Abstract

**Background and objectives**: Peoples’ attempts to lose weight by low calorie diets often result in weight gain because of over-compensatory overeating during lapses. Animals usually respond to a change in food availability by adjusting their foraging effort and altering how much energy reserves they store. But in many situations the long-term availability of food is uncertain, so animals may attempt to estimate it to decide the appropriate level of fat storage.

**Methodology**: We report the results of a conceptual model of feeding in which the animal knows whether food is currently abundant or limited, but does not know the proportion of time, there will be an abundance in the long-term and has to learn it.

**Results**: If the food supply is limited much of the time, such as during cycles of dieting attempts, the optimal response is to gain a lot of weight when food is abundant.

**Conclusions and implications**: This implies that recurring attempts to diet, by signalling to the body that the food supply is often insufficient, will lead to a greater fat storage than if food was always abundant. Our results shed light on the widespread phenomenon of weight gain during weight cycling and indicate possible interventions that may reduce the incidence of obesity.

## Abstract (precis)

Repeated dieting may lead to weight gain because the brain learns that the food supply is unreliable. Animals respond to food shortage by storing fat. Our model of learning shows that if the food supply is restricted (as in dieting) an optimal animal should gain excess weight between diets.

## INTRODUCTION

Overweight and obese people are frequently able to lose weight but are unable to maintain such losses long term [1], which is why a large proportion of individuals are on diets at any given time [2]. Repeated weight loss and gain are referred to as yo-yo dieting or weight cycling [2]. Whilst most people can lose weight during diets, weight gain between diets is proportional to the weight lost [3] and may even lead to new weight gain in the long term [4–7]. Whilst weight cycling *per se* is not associated with health issues [8, 9], the weight gain has many health implications [10]. There are many mechanisms underpinning eating behaviour that may contribute to weight gain [11]. Some research has focussed on the physiological mechanisms that cause long-term weight gain in response to repeated dieting attempts, such as changes in the production of regulatory hormones [5, 7], which may shift the body’s response to signals from adipose tissue [12].

Whilst it is essential to understand the mechanisms, the search for treatments for obesity will involve achieving a holistic understanding of regulatory systems. A descriptive model that mimics the cycling phenomena [13] assumes that weight gain stops at some maximum and weight loss stops at some minimum. But this model does not elucidate why, in evolutionary terms, a system would be designed as it is supposed. An evolutionary perspective can help to elucidate the causes of being overweight and obese [14]. Evolutionary arguments centre around the usefulness of fat as a source of energy under food shortage and the costs of carrying stored fat [15]. Models of adaptive behaviour that consider fat as a means to reduce the risk of starvation have been highly successful at predicting energy storage in animals [16–23]. These models typically do not try to capture the complexities of physiological and psychological mechanism that control eating [11, 24, 25], but provide functional explanations for the values of states that arise from such mechanisms, such as the quantity of energy that is stored [26]. Evolutionary approaches to understanding obesity [27, 28] typically assume that humans will have physiological and cognitive systems that evolved in natural (ancestral) environments and have not changed since then, and we know that maladaptive behaviours of various kinds can emerge from strategies that are adaptive in natural environments [29]. Evidence suggests that energy use in western environments is similar to that for hunter-gatherers [30], suggesting that excessive food consumption rather than sedentary lifestyles causes obesity.

Humans appear to have sophisticated controls on fat storage that act to maintain weight at some target, but the variation in body weight within populations indicates that this target must differ between individuals [31]. It has not been fully elucidated why individuals might differ in this way. Existing data show that whilst a significant proportion of the variation in body mass index is attributable to genetic factors [32], there are strong effects of socioeconomic factors [33]. This indicates that learning may play an important role in determining the individuals’ targets. Here, we assess how weight gain after dieting attempts could be an adaptive response involving learning about the environment. Our model provides proof of the concept that weight gain may be a response to an environment to which the evolved subconscious system for controlling energy storage is no longer adapted.

## THE MODEL

We assume that humans have evolved in environments where the food supply fluctuates between limited and abundant, but also that there are times, years or seasons, where the proportion of time that food is abundant is greater or lesser [30, 34, 35]. The current level of food availability is therefore not sufficient to infer the long-term food availability. It is a ubiquitous feature of natural environments that food availability varies over time and shows such positive autocorrelation and our formulation captures this in the simplest possible way. We model a hypothetical animal that uses energetic reserves to meet all its needs and tries to learn about the long-term food availability from observing the short-term fluctuations. This animal is adapted to conditions over evolutionary history in which the food supply fluctuated. We are interested in the consequences if dieting attempts are interpreted by the subconscious brain as such fluctuations.

### The animal and its environment

We model time as a sequence of discrete epochs in which the animal makes a decision and its state variables may change from one epoch to the next. The animal is characterized by four state variables [36]. The first is its level of energetic reserves *x*. There are two external states: the current food condition *C* where food availability is higher in the rich condition (*C = R*) than the poor condition (*C = P*), and the current state of the world *W* which can be good (*W = G*) or bad (*W = B*), which differ in the average durations of rich and poor periods. The animal knows the current conditions without error, but does not directly know whether the world is good or bad. The final state variable is the animal’s current estimated probability that the world is good (*ρ*). Note that we do not assume that any animal has a perfect system for calculating probabilities, but that evolution has selected for a cognitive system that behaves as though it tracks a probability. At the end of a decision epoch, the world changes from its current state *W* to the alternative state with probability θ_*W*_. When the world is in state *W* conditions change from the current condition *C* to the alternative condition with probability λ_*W,C*_. We fix these probabilities so that conditions are predominantly rich in the good world and often poor in the bad world, and that conditions change much more frequently than the state of the world (θ_*W *_≪ λ_*W,C*_). Examples of food availability over time in good and bad worlds are shown in Supplementary Fig. S1. Each decision epoch the probability that the world is currently good (*ρ*) is updated using Bayes’ rule. Supplementary Fig. S2 illustrates how probabilities are updated for the baseline parameter values.

The aspect of behaviour we are interested in is the proportion of time the animal spends foraging per decision epoch, which we call *f*. Increasing *f* increases the probability of finding food. Poor and rich conditions differ only in the maximum probability of finding food per decision epoch when foraging (*γ_R_* and γ_*P*_, where γ_*R*_ > γ_*P*_); the animal finds food during unit time with probability *γ_C_f*. For computational reasons, there is some variance in the energy content of food items (see Supplementary Appendix) and they contain on average *b* units of energy.

In natural environments, there are a variety of costs of carrying fat reserves. In modelling fat regulation in small birds, it is usual to assume that energy expenditure increases with the amount (and hence weight) of fat carried. It is also often assumed that predation risk when foraging increases with increasing fat load because of decreasing maneuverability [37]. Regardless of the exact cost, some cost needs to be assumed if long-term adaptive fat levels are to be stable [38]. In humans, it seems reasonable to assume that the rate of energy expenditure during activity increases with increasing fat load. This would then impose a cost since increased expenditure requires increased time finding food, resulting in less time that is available to spend on other activities. Our model is based on such a cost. We assume that the animal’s rate of energy expenditure *m*(*x*) increases with energy reserves *x*—representing the energetic costs of carrying fat in humans [39] and animals [40]—according to

(1) $$m(x)=m_{0}\left[1+m_{x}\frac{x}{x_{\max}}\right]$$

where *m_x_* (>0) controls how the cost increases with reserves, and *m_0_* controls the magnitude of costs. For the baseline parameter values (Table 1), this means that an animal with maximum fat stores would use energy at twice the rate of an animal with no fat. A consequence is that the benefit of building up energetic reserves will diminish, so we never predict that stores should be near the maximum. We set other parameter values so that the expected net rate of energy gain at *f = *1 in bad conditions is slightly positive; thus, there is a risk of starving to death, but animals are expected to survive sufficiently long that the model makes clear predictions about the effects of other parameters.

**Table 1.** Parameters and variables in the model and their baseline values

Symbol	Description	Value
*Individual*		
*x*	Energy reserves	0 – *x_max_*
*ρ*	Probability that world is good	0 ≤ *ρ* ≤ 1
*x_max_*	Maximum level of energy reserves	100
*V*	Value of the animal’s life	*V* ≥ 0
*f*	Intensity of foraging	0 ≤ *f* ≤ 1
*m_0_*	Magnitude of energy use	0.5
*m_x_*	Dependence of energy use on reserves	1
*m_f_*	Dependence of energy use on activity	0
*m_x,f_*	Dependence of the cost of reserves on activity	0
*Environmental*		
*b*	Mean energy in food items	5.5
*μ*	Probability of mortality per decision epoch	0.00001
*θ_W_*	Probability that world *W* changes to other world	*θ_B_* = 0.0001, *θ_G_* = 0.0001
*λ_W,C_*	Probability that world *W* in condition *C* changes to the other condition	*λ_B,P_* = 0.05, *λ_B,R_* = 0.05
		*λ_G,P_* = 0.1, *λ_G,R_* = 0.02
*t_W,C_*	Mean number of decision epochs for which world *W* stays in condition *C* (*t_W,C_* = 1/*λ_W,C_*)	*t_B,P_* = 20, *t_B,R_* = 20
		*t_G,P_* = 10, *t_G,R_* = 50
*γ_C_*	Probability of finding food in condition *C* per unit time spent foraging	*γ*_P_ = 0.3, *γ*_R_ = 0.7

We assume that there are two sources of mortality [41]. If the energy reserves of the animal reach *x* = 0, the animal dies of starvation. During each epoch, there is also a probability *μ* of death from external sources that is independent of state and behaviour. We assume that the time that the animal does not spend foraging is invested in increasing its reproductive success, such as in courting potential mates. This reproductive payoff is instantaneous and subject to diminishing returns so that foraging for a proportion *f* of a single decision epoch increases the animal’s lifetime reproductive success by $\sqrt{1-f}$. There is therefore a trade-off between immediate investment in reproduction and increasing the future investment by finding food to increase the expected lifespan. A strategy specifies how the value of *f* depends on the three state variables *x*, *ρ* and *C* (*W* is not directly known). The optimal strategy f* maximizes the total lifetime reproductive success of the animal. Under this strategy, the proportion of time spent foraging when the combination of state variables is (*x, ρ,C*) is f*(*x, ρ,C*). We use standard methods of stochastic dynamic programming [36] to find this strategy. See Supplementary Appendix for full details.

### Cost of being active

Thus far, we have assumed that the rate of energy use is the same whether the individual is foraging or not, but fitness-promoting activities may be sedentary (e.g. grooming) or active (e.g. singing). To allow for the dependence of energy use on activity, we set the rate of energy expenditure to be

(2) $$m(x,f)=m_{0}\left(m_{f}f+\{1-m_{f}\}\right)\times \left[1+\left(m_{x,f}f+\{1-m_{x,f}\}\right)m_{x}\frac{x}{x_{\max}}\right]$$

where *m_f_* controls the dependence of energy expenditure on activity and *m_x,f_* controls the dependence of the costs of energy reserves on energy use when active (i.e. the interaction). Note that if *m_f _*=*m_x,f_* =0 we recover Equation (1). If all else were equal, the extra costs of activity would decrease average energy expenditure (because *f *≤**1), and so average costs and type of costs would be confounded in any comparison. To minimize the effect of average costs, we adjusted the value of *m_0_*. The approximate mean value of reserves under normal conditions for baseline parameter values (Table 1) is 25, so the average energy use will be around $\frac{1}{2}\left[1+\frac{25}{x_{\max}}\right]=\frac{5}{8}$. We took average *f* to be 0.5, and so use a value of *m_0_* given by

(3) $$m_{0}=\frac{\frac{5}{8}}{\left(\frac{m_{f}}{2}+\{1-m_{f}\}\right)\left[1+\left(\frac{m_{x,f}}{2}+\{1-m_{x,f}\}\right)m_{x}\frac{25}{x_{\max}}\right]}.$$

### Assessment of behaviour

The dynamic programming procedure calculates the reproductive value of the animal in all states *V*(*x, ρ,C*), which is the expected contributions to reproductive success before death. We use *V* to assess the strength of the urge to add to fat stores by calculating the risk that would be tolerated to gain the equivalent of two items of food. Specifically, we calculate the extra mortality risk *μ*′ at which the animal is indifferent between its current situation and gaining 10 extra units of reserves at risk *μ*′. This mortality risk satisfies

(4) (1−μ')V(x+10,ρ,C)=V(x,ρ,C).

Rearranging gives

(5) $$\mu^{\prime }=1-\frac{V(x,\rho ,C)}{V(x+10,\rho ,C)}.$$

We calculate the average amount of energy stored when following the optimal strategy in four conditions. First, under normal conditions in the good world with conditions changing between poor and rich according to the values of *λ_G,P_* and *λ_G,R_*. Second, for constant rich conditions, which we refer to as ‘glut’. Third, when conditions switch slowly between poor and rich, referred to as ‘slow diet’. Fourth, when conditions switch rapidly between poor and rich, referred to a ‘quick diet’. Thus, we simulate different dieting patterns. We are interested in the predicted energy storage and the belief that the world is good (*ρ*) under these four conditions.

## RESULTS

**Figure 1.**
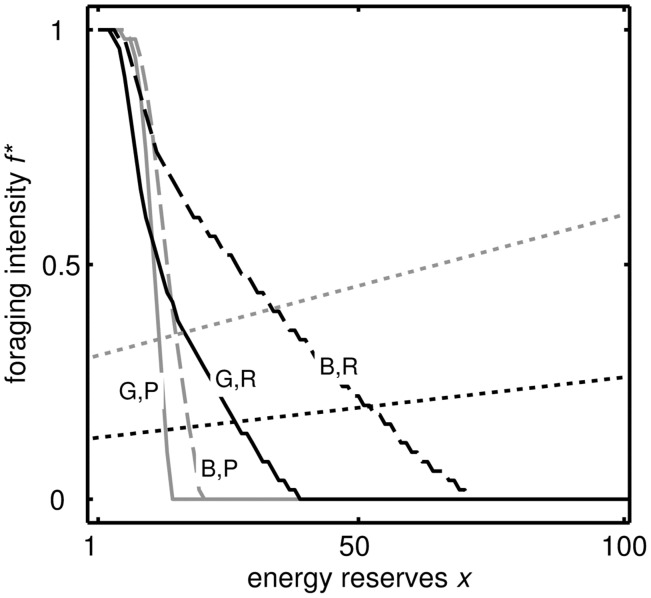
Optimal strategy of foraging intensity f* for reserves *x* and poor (‘*P*’, grey) and rich (‘*R*’, black) conditions for *ρ *= 0 (‘B’,dashed) and *ρ *= 1 (‘G’, solid) for the baseline parameter values shown in Table 1. *f* * changes smoothly for intermediate values of ρ (not shown). Dotted lines indicate the value of *f* necessary to maintain a constant level of reserves long-term in rich (black) and poor (grey) conditions. Hence, where the strategy lines of the same shade intersect the dotted lines is the target level of reserves. The target level of reserves in rich conditions is higher in the bad world than the good world

Figure 1 shows the optimal strategy for the baseline parameter values (Table 1). Generally, the optimal foraging rate f* is higher in the bad world because the animal must attempt to have greater insurance against the risk of going without food and starving. In both worlds, f* is greater at low reserves in poor conditions than in rich conditions because it is crucial to find food before starvation, whilst at high reserves f* is greater in rich conditions than in poor conditions (even in the good world) because it is worth trying to build up the insurance when food is abundant (for more exploration of conditions see [42]). The target level of reserves in rich conditions is higher in the bad world than in the good world because more insurance is needed as the period of food shortage is likely to be longer.

**Figure 2.**
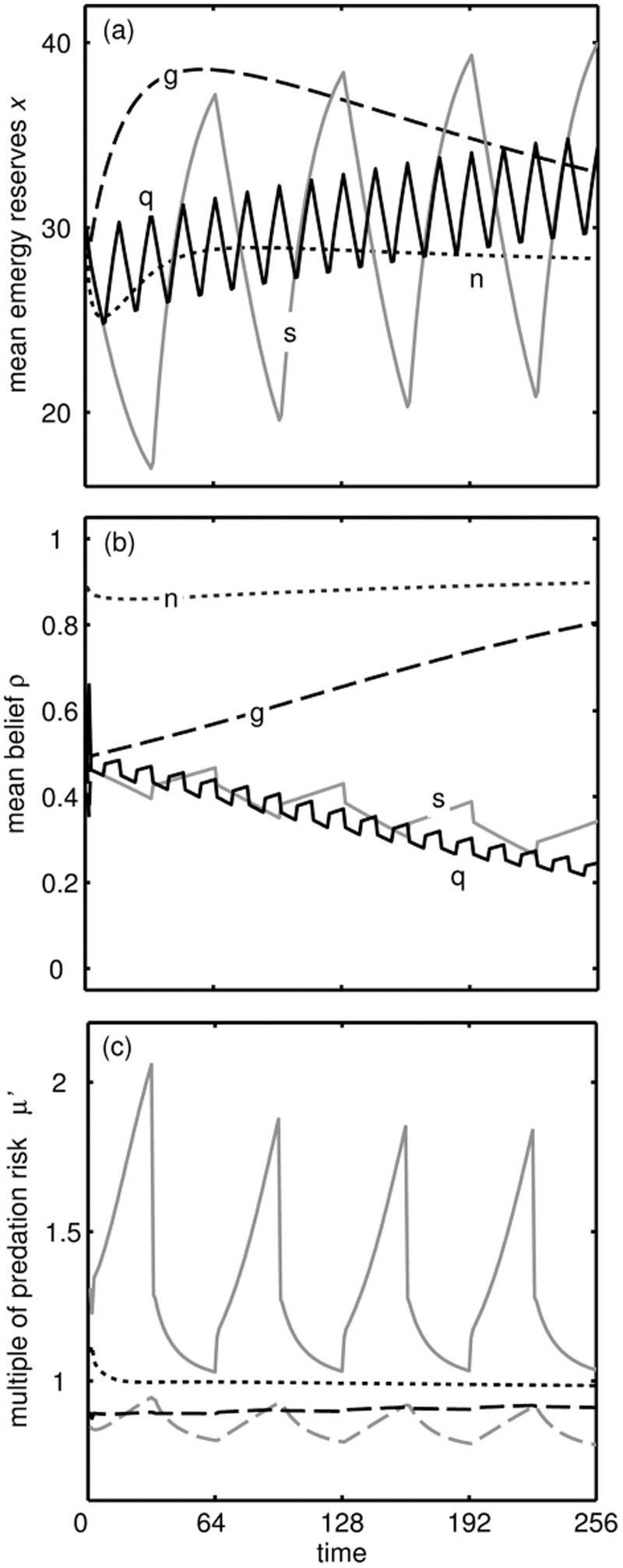
Effect of three ‘treatments’ compared to control conditions. (a) Mean energy reserves *x* over time when conditions always rich (‘glut’: *g*, dashed line) or when conditions switch between poor and rich every 32 epochs (‘slow dieting attempts periods’: *s*, solid grey line), or when conditions change between Poor and Good every 8 epochs (‘quick dieting attempts’: *q*, solid black line), compared to the mean across Poor and Rich conditions in the Good world (‘control’: *n*, dotted line). (b) Belief that the world is Good *ρ* for the same period and treatments. Under normal conditions ρ settles down at a high level, whereas during a glut conditions are always rich so learning is slower as *λ_B,R _*≈ *λ_G,R_*. (c) Selective pressure to eat food. We plot over the course of the slow dieting periods the mortality risk that would be tolerated to get 10 units of energy *μ’*, as a multiple of what *μ’* would be tolerated under control conditions (solid grey line), and for comparison the same metric for reserves in the control conditions and belief under diet conditions (dashed black line), reserves under diet conditions and belief under control conditions (dashed grey line), and reserves and belief under constant rich conditions (dotted line)

Constant glut conditions lead to greater energy reserves than under normal conditions, but the response to periods of poor conditions leads to overcompensation when conditions become rich (Fig. 2a). This results in greater energy reserves after dieting attempts than in constant glut conditions. This occurs because the animal becomes convinced that the world is bad (Fig. 2b) and that it must take advantage of rich conditions whilst they last. If conditions fluctuate quickly, reserves are lower in the short term (Fig. 2a) but the animal becomes more convinced the world is bad over the longer term (Fig. 2b). The extra mortality risk that would be tolerated to get two food items is plotted for as a function of current reserves when following the slow diet (Fig. 2c). Because the animal is convinced the world is bad, it is willing to risk up to 2× greater than in a constant glut when reserves become low. However, this increase depends on the combination of reserves being low and the belief that the world is bad: lower values of *μ*′ than for glut conditions are predicted at low reserves and believing the world is *good* (grey dashed line) and believing the world is bad at *high* reserves (black dashed line).

The reasons for the weight gain after the period of poor conditions can be understood by considering the optimal strategy (Fig. 1). When the individual has a period of poor conditions then switches to rich, it ‘believes’ that the world is bad, so the gain in reserves is greater than it would have been if conditions were always rich. Thus, the gain in weight after repeated dieting comes about because an animal with high reserves should forage more in rich conditions especially when it believes that there is a strong possibility that conditions will turn poor. The reserves stored under dieting approach an asymptote over a longer period of time, whereas under constant glut they drop down to that stored under normal conditions (Supplementary Fig. S3) because the individual becomes convinced the world is good so there is no need to store much energy.

**Figure 3.**
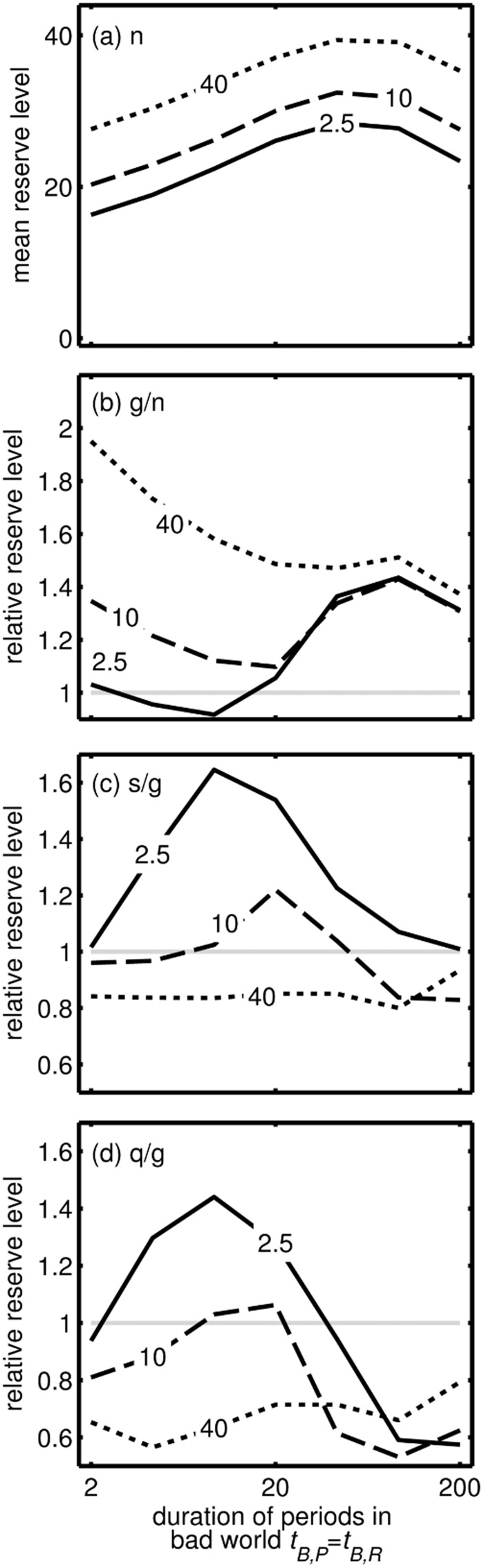
Effect of mean duration of both poor and rich periods in the bad world (*t_B,R _= *1/*λ_B,R _*=* t_B,P _= *1/*λ_B,P_*, x-axis) and mean duration of Poor periods in the good world (*t_G,P_*_,_ shown on lines) on (a) mean reserve level in the good world, (b) extra reserves storage during a glut as a proportion of reserves under normal conditions, (c) extra reserves storage after a slow dieting attempt as a proportion of reserves under glut conditions, (d) extra reserves storage after a quick switching dieting attempt as a proportion of reserves under glut conditions

The mean reserves stored in the good world increases with the duration of bad periods and with the duration of periods in the bad world (Fig. 3a), due to the insurance effect. To illustrate this effect, we present the optimal strategy in Supplementary Fig. S4 for 9 of the 21 parameter value combinations used to make Fig. 3. Reserves in a glut are greater relative to under normal conditions when poor periods in the good world are longer (Fig. 3b). Hence, the greatest gain after dieting attempts, relative to glut conditions, is when poor conditions are short in the good world (Fig. 3c) because this causes a greater difference in the target level of reserves in rich conditions between the bad and good world (cf. Supplementary Fig. S4b and h). After 256 decision epochs, lower reserves are stored if dieting fluctuations are quicker (cf. Fig. 3c, 1) for most situations and exceed reserve level expected in a glut only if poor periods are very short in the good world.

**Figure 4.**
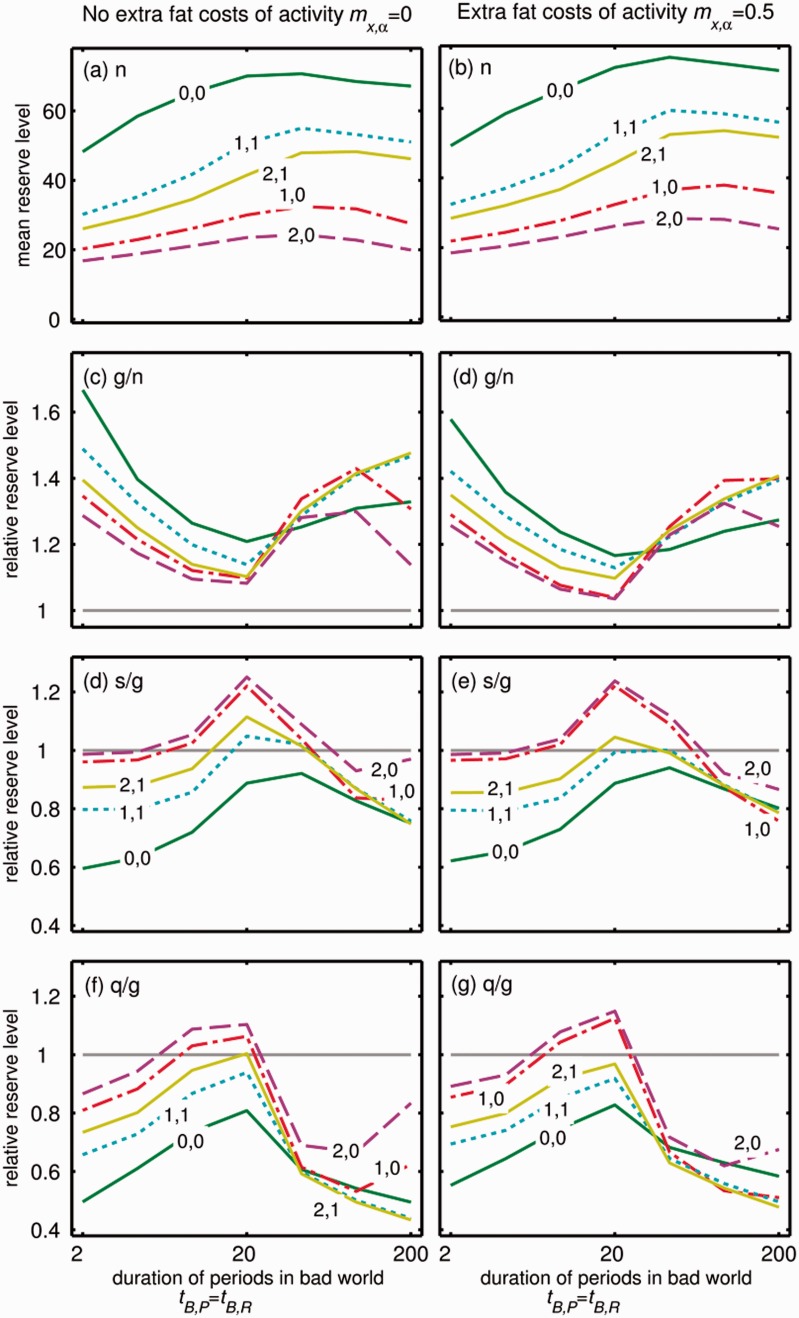
Effect of mean duration of both poor and rich periods in the Bad world (*t_B,R _= *1/*λ_B,R _*=* t_B,P _= *1/*λ_B,P_*, x-axis) on energy storage for 10 realizations of the dependence of energy use on reserves and activity. Panels show (a, b) mean reserve level in the good world, (c, d) extra reserves storage during a glut as a proportion of reserves under normal conditions, (e, f) extra reserves storage after a slow switching dieting attempt as a proportion of reserves under glut conditions, (g, h) extra reserves storage after a quick switching dieting attempt as a proportion of reserves under glut conditions, and (a, c, e, f) no extra costs of energy reserves when active *m*_*f*_ = _**_0, and (b, d, f and g) energy reseves are more costly when active *m*_*f*_ = _**_0.5. Lines are shown for various values of *m_x_* (first value: 0, 1 or 2) and m*_x,f_*(second value: 0 or 1)

One explanation for difficulty in losing weight is that lighter bodies require less energy so food consumption needs to progressively reduce [7]. In Fig. 4, we show the effect of changing the magnitude of the dependence of energy expenditure on the level of reserves. In all conditions more fat is stored in constant glut compared than under normal conditions. Although larger values of *m_x_* than unity tend to either decrease (*g/n*) or increase (*s/g, q/g*) relative reserve levels, the overall pattern is unchanged. However, when *m_x_* is zero—meaning that energy use does not increase with energy storage—we do not predict dieting to cause weight gain (*s/g *<**1 and *q/g *<**1), suggesting that the energetic cost of fat storage is essential to the increase in body weight due to weight cycling.

The optimal strategy is influenced by changing the various costs (*m_x_, m_f_, m_x,f_*, see Supplementary Fig. S5). The effect of dieting is considerably weaker when *m_x,f _*>_**_0 because the extra cost of activity means that the animal gains more reserves in rich conditions. However, the effect is stronger in the good world, and so results in less of a difference between glut and dieting conditions. *m_f_* has very small effects on predicted energy storage (cf. Fig. 4, left and right panels) because the individual can decrease costs in poor conditions by being inactive, which reduces the advantage to storing fat, and this cancels out the selective pressure to store more fat in response to increased costs. There is discrepancy between the level of reserves that individuals should try to store (‘target’) and the reserves that can be built up (‘realized’), which differ due to stochasticity (Supplementary Fig. S6), and the difference depends on the types of costs (Supplementary Fig. S6e and f). Note that in all cases, the discrepancy in dieting conditions is much smaller than in glut conditions. Based on the discrepancy between the target and the realized state, the urge to eat strongest when the rate of energy use is constant (0, 0 lines in Fig. 4) or increases with reserves and this is at a greater rate when foraging (1,1). The urge to eat will be weakest when the rate of energy use only depends on reserves, but strongly (2,0). Again, the effect of an overall cost of foraging (*m_f_*) is small and constant across other costs (cf. Supplementary Fig. S6e and f).

## DISCUSSION

Weight cycling is common in people that are attempting to lose weight, but many people gain weight in the long term. The functional reasons that our energy storage systems might respond in this way to dieting attempts has not been elucidated. We have used a simple generic model of feeding to demonstrate how a reserve-control system following an ecological rational strategy [43] could cause weight gain over the long-term if periods of food shortage are frequent even if they are associated with short-term weight loss. Our work therefore proposes a potential cause of the association between weight cycling and weight gain [5, 44, 45]: that dieting attempts *cause* weight gain via providing (misleading) information about the environment to the subconscious systems that control body mass. That is, even in the ‘constant glut’ [10] conditions in the developed world where food is always abundant, the subconscious decision-making systems that underpin our behaviour may interpret dieting attempts as indicative of an environment with common food shortages, and this triggers the (previously) appropriate behavioural responses.

Our model predicts that energy reserves should respond to repeated attempts to diet by weight cycling and becoming greater from one cycle to the next. The more reliable food was when the world was good, the greater the relative fat storage during repeated dieting attempts is predicted to be because these dieting attempts cue that the world is more likely to be bad. Thus, the very conditions that cause weight gain initially—a glut of food—causes further weight gain once cyclical dieting begins. There is evidence that among weight cycling, people those who switch between dieting and binge-eating more frequently gain more weight [45]. By contrast, we found that quick oscillations tended to lead to less weight gain for the period we studied, but over the longer term, the duration of dieting periods has little effect on the average energy storage (Supplementary Fig. S3). We concentrate on outcomes after a relatively short period of dieting (256 time steps, Figs 2 and 3), partly because people not only do not diet forever but also energy storage tends to level off (Supplementary Fig. S3). We note that our model predicts that fat storage under constant glut conditions that persist for a long time will actually not be substantially greater than under normal conditions (Supplementary Fig. S3), suggesting that the abundance of energy-rich food is not a complete explanation for the obesity epidemic. Fat storage over a long period of dieting attempts will be greater than under constant glut conditions, implying a critical role of informational constraints and learning.

Our results suggest that the magnitude of the weight gain between diets will depend on the cost of the non-foraging activity. Our rescaling (by adjusting *m_0_*) means that we compare predictions depending on the *relative* cost of the other activity. When *m_f_* and *m_x,f_* are small foraging is much more costly than other activities; when unity other activities are equally costly. For some species and situations, the activities that enhance reproduction may be energetically inexpensive, such as grooming in primates. In other cases, activities essential to reproduction may be equally as energetic as foraging, such as maintaining a territory. It is difficult to know what best applies to humans. However, it may be possible to quantify the relative costs of foraging across species, which would offer possibilities for testing our predictions. Foraging may be relatively more costly than non-foraging in a small bird (i.e. small *m_f_*) compared to a rodent (i.e. large *m_f_*). If we could expose laboratory birds (e.g. zebra finch) and rodents (e.g. mice) to a yo-yo diet regime, we would predict that the rodent would gain more weight. A very large-scale project could try to estimate our cost parameters (*m_x_*, *m_f_*, *m_x,f_*) for several populations or closely related species in order to assess the responses to ‘dieting’ and then measure the target and/or realized level of reserves (Supplementary Fig. S6).

Lowe [3] argues that yo-yo dieting does not cause weight gain but is merely a correlate of the potential for weight gain, which may arise if people who know they often overeat take steps to avoid weight gain. This argument is based on the assumption that causation is one way, but our perspective shows that causation may be two way between food restriction and overeating, leading to a spiral of dieting and weight gain. We suggest that the interpretation of data is hampered by a lack of robust theory, and hope that our work may cause a re-evaluation of observations of weight cycling. For instance, Lowe [3] suggests that weight regain is *caused* not by dieting but by increased binge eating and increased reward value of food. From our perspective, these are proximate mechanisms that implement the behavioural strategy that we have identified; thus both explanations can be true.

Not all individuals acquire excess weight after dieting [46]. Our results suggest that variation among individuals could occur if people have different subconscious expectations of the pattern of food availability (Fig. 3c and d). For instance, weight cycling does not promote extra weight gain if the system ‘expects’ conditions to change very slowly or very rapidly when the world is bad. If such ‘expectations’ were determined by natural selection in different environments and encoded in genes, then this effect may underlie effects of ethnicity on the risk of obesity [33, 47]. On the other hand, this ‘expectation’ may be learnt during a lifetime, which may underlie the effects of age on the apparent heritability of obesity [32]. Furthermore, this provides a possibility for testing our predictions: if young mice occasionally experience periods where food is restricted but is available with various rates of fluctuations (e.g. every other hour; every other day) then when older they should show different responses to intermediate frequencies of food restriction. Specifically, those who were exposed to an intermediate rate of fluctuations may gain the most weight (peaks in Fig. 4), and those used to constant glut conditions would gain more weight relative to control individuals than those subject to occasional food shortage when young (cf. different lines in Figs. 3 and 4).

Experiments that use various protocols of food restrictions could be used to assess the predictions around foraging intensity (Fig. 1; Supplementary Fig. S5), provided there was an appropriate continuous measure of the behaviour of subjects. Since our predictions are state dependent, repeated measures of the same individuals after their fat stores have been manipulated through food restriction or gluts would provide a powerful test. For instance, the crossover points in the strategies mean that at low reserves, we predict higher intensity foraging when food is scarce (e.g. low fixed ratio schedule) than abundant, and the converse when at high reserves. A more challenging experiment could try to manipulate the subjects’ beliefs about not only current conditions but also the ‘’world’: the long-term conditions. Under some parameter combinations (e.g. Supplementary Fig. S4b), we predict that there will be a crossover in foraging intensity when food is currently abundant: at low reserves subjects should show lower intensity foraging if the world is bad but the converse when at high reserves.

The additional risk of mortality that would be incurred to obtain food can be seen as a surrogate for the strength of motivation to eat. Our results on this risk explain why people’s motivation systems strongly push them to eat high calorie food, and why this urge will be especially strong during a diet [48]. Interestingly, we predict that this urge will not gradually diminish over dieting attempts (although calories consumed will be lower) despite weight being gained, because the system becomes more and more convinced the world is bad. People who attempt to diet for a very long time will not continue to gain weight but reach an asymptote (Supplementary Fig. S3), seemingly much higher than those who never diet (constant glut). Real people are much more complex than our model, but it seems likely that people who have been dieting for a long time may benefit from trying to maintain their body weight for some time rather than reduce calorie intake, to ‘convince’ their regulatory systems that the food supply is reliable.

Our cognitive systems will have evolved to reflect the fact that current conditions are informative of future conditions (i.e. the world is temporally positively autocorrelated) [49]. This is a contrast effect [50], a seemingly irrational behavioural phenomenon seen in many animals [51–53], including humans [54] which can arise due to uncertainty about the long-term state of the world [55], and could underlie several other psychological phenomena [29]. Current conditions in the developed world are constant glut [10], but any uncertainty could make people gain further weight because learning about food availability from dieting attempts alters expectations about food availability in the future. That optimal behaviour depends on future expectations is well established [41], but weight gain between diets is another possible example of behaviour being affected by past experience in seemingly irrational ways [56].

We cannot capture all the complexities of weight cycling in a simple model, so we assume that there are two levels of food availability and study a single cycle, finding when the level of fat should be greater at the end of the cycle than it would otherwise have been. In reality, people are learning over the long term. However, we find that the weight gain slows as more fat is stored (Supplementary Fig. S3), which is consistent with the observation that obese people do not gain further weight as a result of dieting [7], so we expect that a more long-term model would not lead to further insights. Our model only captures the function of fat storage, and we have not attempted to specify the psychological or physiological mechanisms that bring it about; one possible mechanism is an alteration of the sensitivity of anabolic responses to adiposity signals [12].

Further developments of our model could include decision-making about how much lean mass should be stored and when protein might be catabolized for energy, as we have shown this flexibility may affect decisions about fat storage [57, 58]. However, even our simple model demonstrates the principle that understanding weight gain during yo-yo dieting does not require recourse to explanations based around the feeding control system malfunctioning [1, 11] or being overwhelmed by modern food stimuli [10, 11]. The feeding system could be functioning perfectly, but uncertainty about the food supply triggers the adaptive response to gain weight.

## Supplementary Material

Supplementary Data
