# Supplementary material for: An adaptive response to uncertainty can lead to weight gain during dieting attempts
Source: Evol Med Public Health. 2016 Dec 5;2016(1):369–80. doi: 10.1093/emph/eow031 (PMC5139007; doi:10.1093/emph/eow031)
Supplement: Supplementary Data [file supp_2016_1_369__index.html]

An adaptive response to uncertainty can lead to weight gain during dieting attempts — Supplementary Data 

# An adaptive response to uncertainty can lead to weight gain during dieting attempts

## Supplementary Data

files

- Supplementary Data - doc file
