# Supplementary material for: An adaptive response to uncertainty can lead to weight gain during dieting attempts
Source: Evol Med Public Health. 2016 Dec 5;2016(1):369–80. doi: 10.1093/emph/eow031 (PMC5139007; doi:10.1093/emph/eow031)

**ONLINE APPENDIX**

**An adaptive response to uncertainty can lead to weight gain during dieting attempts**

**A. D. Higginson, J. M. McNamara**

**Evolution, Medicine, and Public Health**

Here, we provide a complete description of the model implementation. The overall framework can be summarized as follows. At a decision epoch reserves are assumed to take values in the range$x=0, 1, 2, \cdots,s$, the conditions *C*  are either rich (*C=R*) or poor (*C=P*), and the world *W* can be either good (*W* = G) or bad (*W* = B). The animal knows *x* and *C* but does not know *W*, instead storing the probability *ρ* that the world is good. At each decision epoch the animal chooses the proportion of time foraging. The proportion of time foraging is allowed to depend on *x*, *ρ* and *C*. A strategy *f* specifies this dependence; under *f* the proportion of time foraging when reserves are *x*, the probability that the world is good *ρ*, and the food conditions are *C* is$f(x,\rho,C)$. Let $V_{f}(x,\rho,C)$denote the expected total future lifetime reproductive success from the current epoch of an individual that is initially in state $(x,\rho,C)$ and follows strategy *f* until it dies. Let

$V *(x,\rho,C)=\max_{f}V_{f}(x,\rho,C)$,

where the maximum is taken over all strategies *f*. If a strategy *f** satisfies $V_{f*}(x,\rho,C)=V *(x,\rho,C)$for all combination of states $(x,\rho,C)$then we refer to *f** as an optimal strategy. This strategy then maximises the total lifetime reproductive success of the animal for all possible initial states. Standard results from the theory of Markov decision processes show that such a strategy exists (Puterman 2005). In this appendix we detail how the optimal strategy can be found.

Model details are as follows. If the proportion of time spent foraging in each decision epoch *t* is *f*, then the probability of finding an item of food during this time interval is *γ_C_f,* To avoid potentially problematic grid effects [36], we assume that food items are of two types (type *j* = 1 or 2) with relative abundance *β_j_* (Σ*β_j_* = 1), and provide a reward of energetic value *r_j_*. Foraging for a proportion *f* of a single decision epoch increases the animal’s lifetime reproductive success by $\sqrt{1-f}$. There is therefore a trade-off between immediate investment in reproduction and increasing the future investment by finding food to increase the expected lifespan.

We assume that the forager pays an energy cost *m*(*x*) to meet its metabolic needs in each decision epoch

$m(x)=m_{0}+m_{x}x$,

where *m_x_* controls how the cost increases with reserves and *m_0_* is the cost at zero reserves. The probability the animal is not killed by a predator before the next decision epoch is $1-\mu$. If the change in reserves results in the new reserves being zero or below, the animal is assumed to have died of starvation. If the new reserves would have been greater than the maximum value of *s*, then reserves are taken to be *s*. Thus if the animal has reserves *x* and forages for a proportion of time *f* its reserves (*x’*) at the next decision epoch given it does not die of predation are

$x'_{0}=\max(x-m(x),0),\text{ with probability }p_{0}=(1-\gamma_{C}f)$,

$x'_{1}=\min(x+r_{1}-m(x),s),\text{ with probability }p_{1}=\gamma_{C}f\beta_{1}$,

$x'_{2}=\min(x+r_{2}-m(x),s),\text{ with probability }p_{2}=\gamma_{C}f\beta_{2}$.

Computations are based on the values $r_{1}=5$, $r_{2}=6$, $\beta_{1}=0.5$, $\beta_{2}=0.5$; so the mean energetic value of a food item is *b*=5.5.

Let $\rho$denote the current probability that the world is good. This probability is updated as follows. The world may have changed before the animal assesses the conditions, so the intermediate probability is

$\hat{\rho}=(1-\rho)\theta_{B}+\rho(1-\theta_{G})$.

The animal knows what the conditions were at the previous decision epoch and now assesses what the conditions currently are. From this and the prior probability$\hat{\rho}$ the posterior probability that the world is good ($\rho'$) is given by Bayes’ rule. If conditions were poor and are still poor:

And similarly,

calculates *ρ*’ if conditions were poor and are now rich; rich and are now poor; rich and are still rich, respectively. See Figure A2 for values for the baseline parameter values (Table 1).

Given these ingredients, the dynamic programming operator *T** can be expressed follows. Let *V* be a function, $V\left(x,\rho,C\right)$, of energy reserves *x* and the probability that conditions are good *ρ* and conditions *C*  satisfying $V\left(0,\rho,C\right)=0$. Then $T *V$is a new function of reserves and environmental conditions that satisfies $(T *V)\left(0,\rho,C\right)=0$for all *W* and $(T*V)\left(x,\rho,C\right)=\max_{f} H(x,\rho,C;f,V)$ for *x* > 0 and all *ρ* and all *C*, where

where *¬C* indicates the other condition (i.e. *R* when *C=P*; *P* when *C=R*).

To find the optimal strategy, we define a sequence of functions $V_{0}, V_{1}, V_{2},\cdots$ iteratively as follows. Initially set $V_{0}\left(0,\rho,C\right)=0$ for all ρ and *C* and $V_{0}\left(x,\rho,C\right)=1$ for all $x>0$ and all ρ and *C*. Given $V_{k}$, set $V_{k+1}=T *V_{k}$. Then the sequence of functions converges pointwise to a limit $V *=\lim_{k\to\infty} V_{k}$ (Puterman 2005). Convergence was judged to have occurred when $\left|V_{k+1}-V_{k}\right|<10^{-6}$, which typically happened within 500 iterations. Any strategy *f** satisfying $H(x,\rho,C;f*(x,\rho,C),V*)=\max_{f} H(x,\rho,C;f,V*)\text{ for all }x>0\text{ and all }\rho\text{ and }C$ (A4)

necessarily satisfies equation (A2), and is hence optimal (Puterman 2005). This process finds the optimal Bayesian strategy *f**(*x*,ρ,*C*). The optimal strategies for nine combinations of switching probabilities are shown in Figure A3.

**Reference**

Puterman M. L. (2005) Markov decision processes: Discrete stochastic dynamic programming. Wiley, New Jersey.

**Figure A1**: Examples of the probability of finding food (*γ_C_*) over time when the world is (a) Bad and (b) Good. The duration of periods between changes in *γ_C_* (‘conditions’) is variable, but *γ_C_* is more often low (poor conditions) when the world is bad than when it is good.


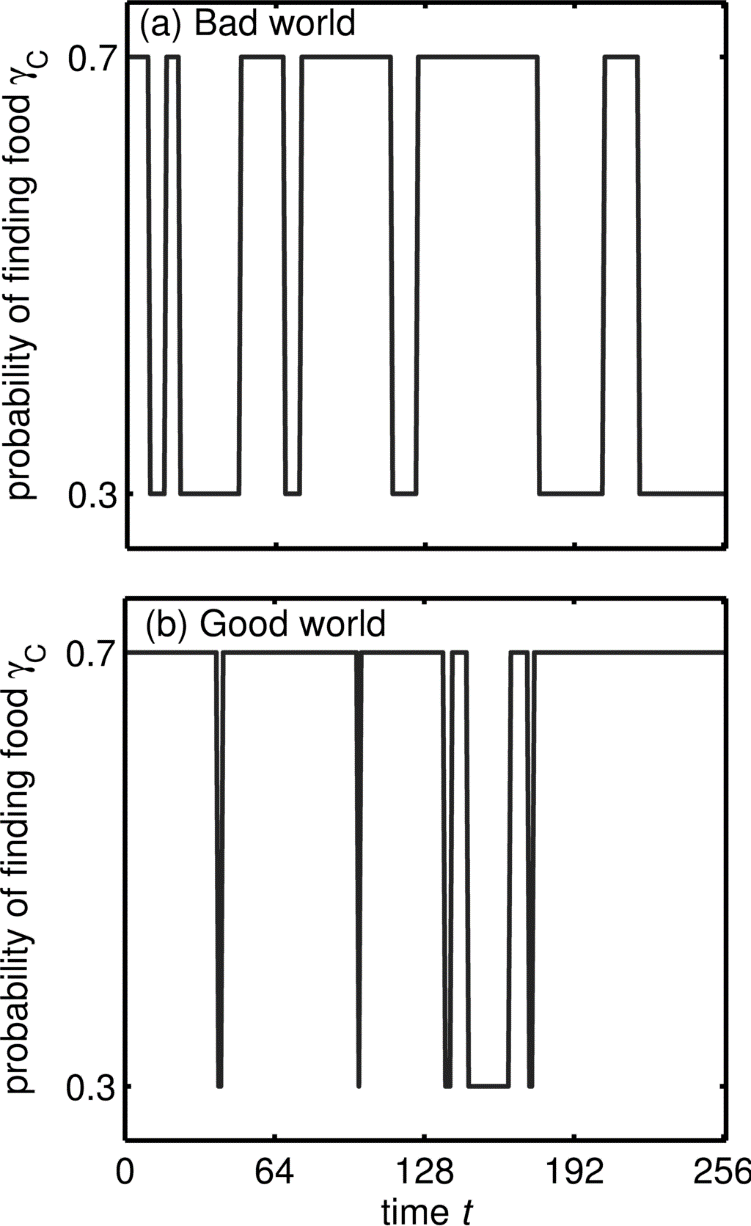


**Figure A2**: Probability *ρ’* that the world is currently good given that the probability at the previous decision epoch was *ρ* and that conditions have changed as indicated on the lines (e.g. P->R indicates that the conditions have changed from poor to rich; R->R indicates that the conditions have remained rich).


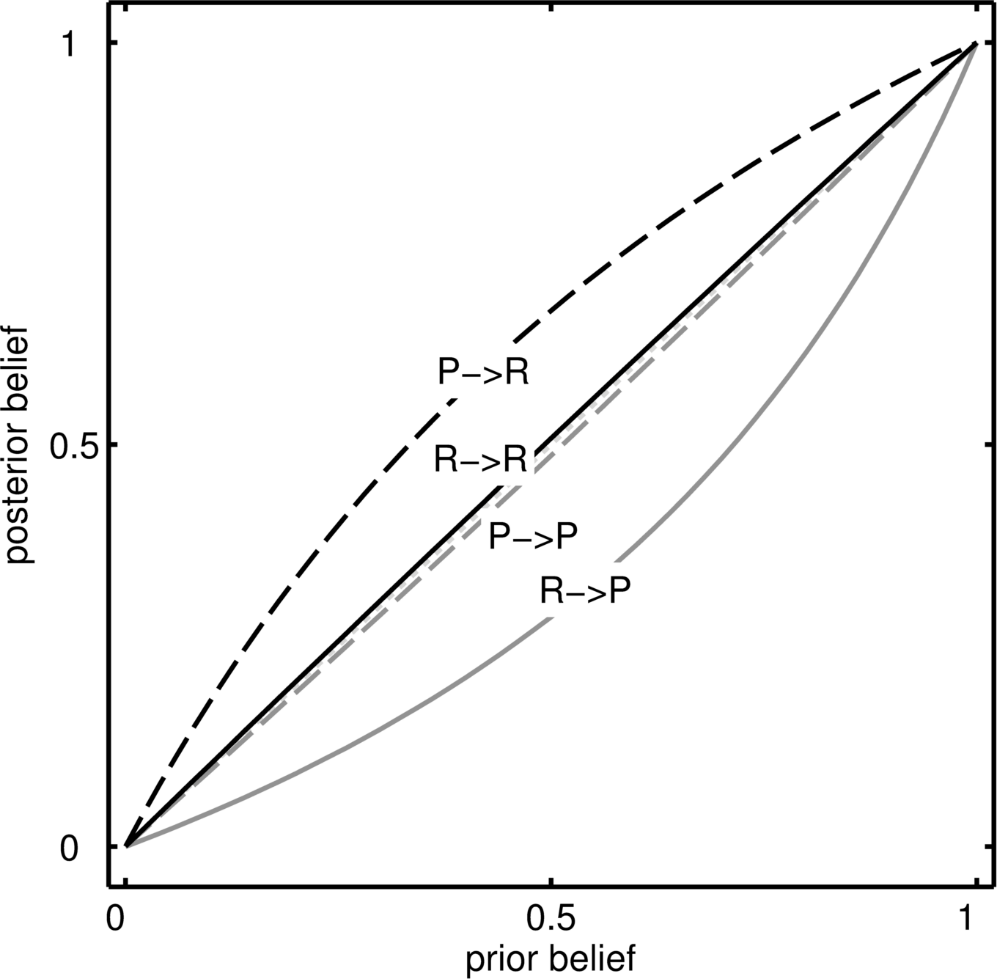


**Figure A3**: Effect of three ‘treatments’ compared to control conditions for a very long period (i.e. as Figure 2 but 2048 time steps). (a) Mean energy reserves *x* over time when conditions always Rich (‘glut’: *g*, dashed line) or when conditions switch between Poor and Rich every 32 epochs (‘slow dieting attempts periods’: *s*, solid grey line), or when conditions change between Poor and Good every 8 epochs (‘quick dieting attempts’: *q*, solid black line), compared to the mean across Poor and Rich conditions in the Good world (‘control’: *n*, dotted line). (b) Belief that the world is Good *ρ* for the same period and treatments. Under normal conditions ρ settles down at a high level, whereas during a glut conditions are always rich so learning is slower as *λ_B,R_*≈*λ_G,R_* but tends towards one, and in the long term energy storage in a glut tends towards that under normal conditions. In dieting conditions *ρ* tends towards zero, and the long term mean is approximately the same for quick and slow fluctuations. The arrow indicates the endpoint of Figure 2 and the point at which the values shown in Figure 3 are calculated.


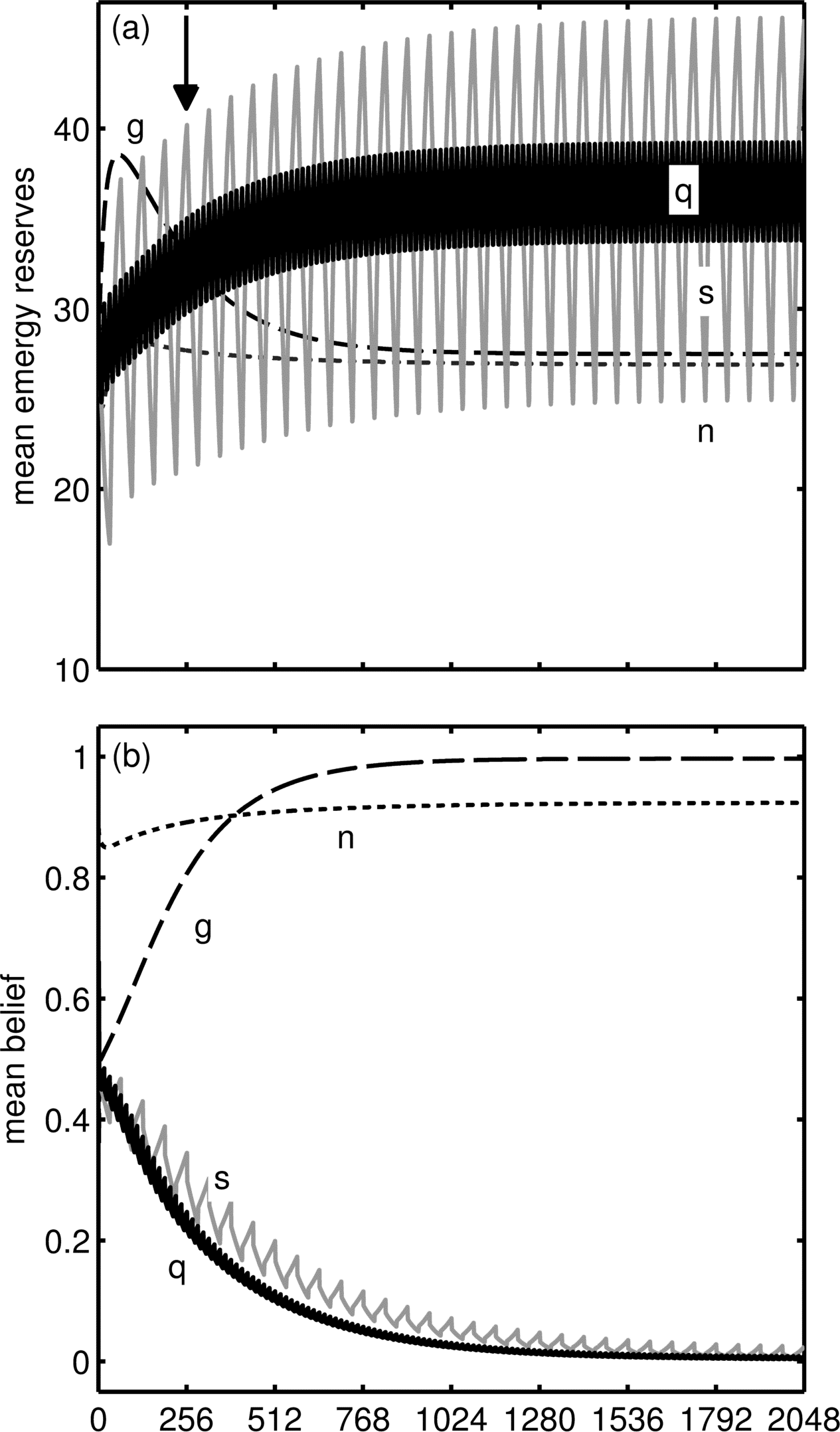


**Figure A4**: Optimal foraging intensity *f** as a function of energy reserves *x* for three durations of rich and poor conditions in the bad world (*t_B,C_*) and three durations of poor conditions in the bad world (*t_G,P_*) as shown on panels, to aid interpretation of Figure 3. Lines are shown for Poor (grey) and Rich (black) conditions, and for *ρ*=0 (dashed) and *ρ*=1 (solid). Other parameter values as shown in Table 1.


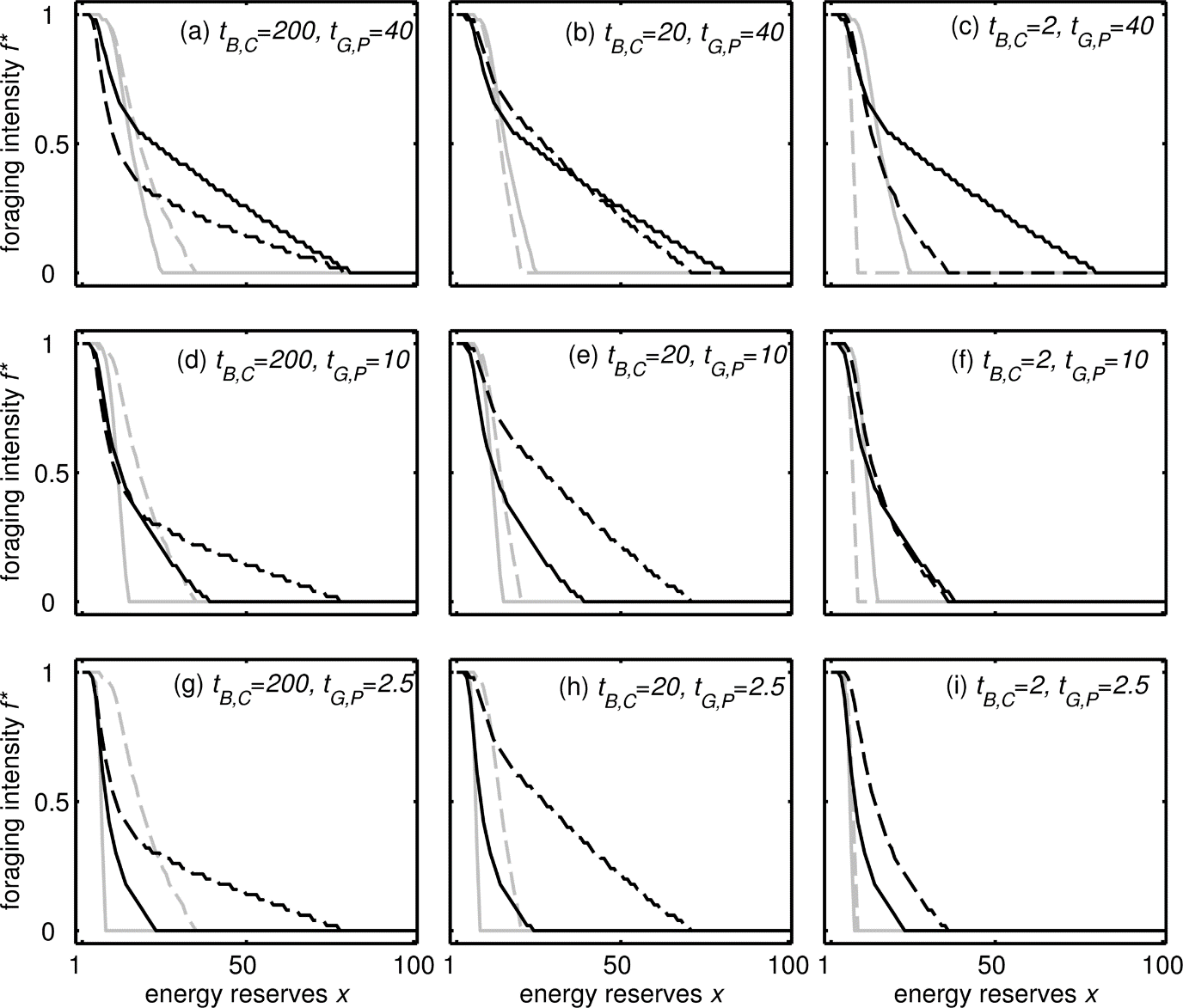


**Figure A5:** Optimal foraging intensity *f** as a function of energy reserves *x* for the types of energy costs (*m_x_*, *m_f_*, *m_x,f_*) shown on panels, to aid interpretation of Figure 4. Lines are shown for Poor (grey) and Rich (black) conditions, and for *ρ*=0 (dashed) and *ρ*=1 (solid). Other parameter values as shown in Table 1.


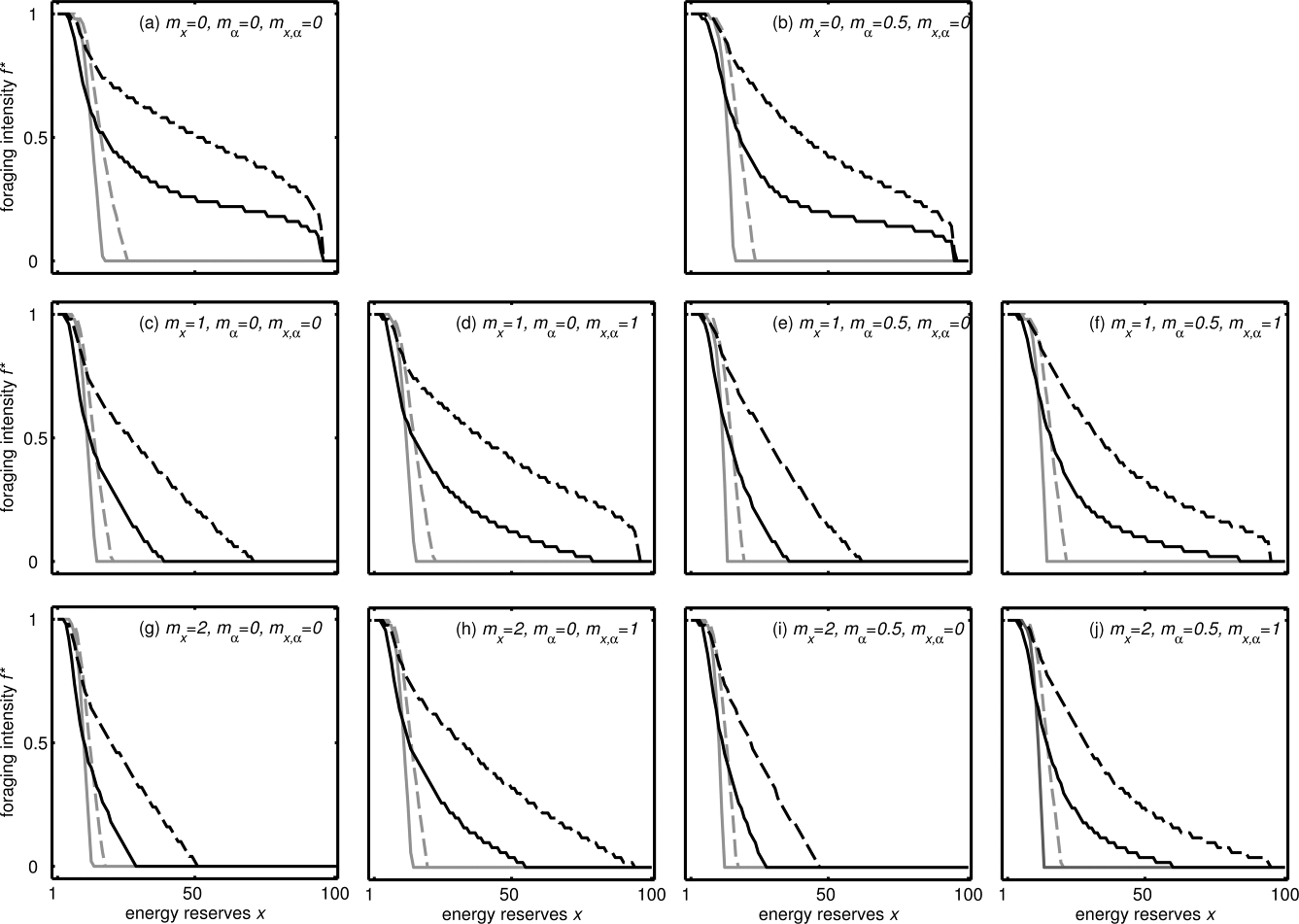


**Figure A6**: Target and realised reserves for various costs (a, c, e: *m_f_*=0; b, d, f: *m_f_*=0.5; *m_x_* and *m_x,f_* as shown on x-axis). (a, b) Target: the level of reserves at which the optimal foraging rate *f**(*x*,ρ,*C*) is expected to result in no change in reserves (*x**). Legend indicates P: poor conditions, R: rich conditions, B: certain world is bad ρ=0, G: certain world is good ρ=1. (c, d) Realised mean reserves after 256 time steps for constant glut (g), slow diet (s), quick diet (q). (e, f) Difference between the realised mean reserves and the target, when the target is the weighted average of the target in the bad and good world, weighted by the probability the world is good after 256 time steps.


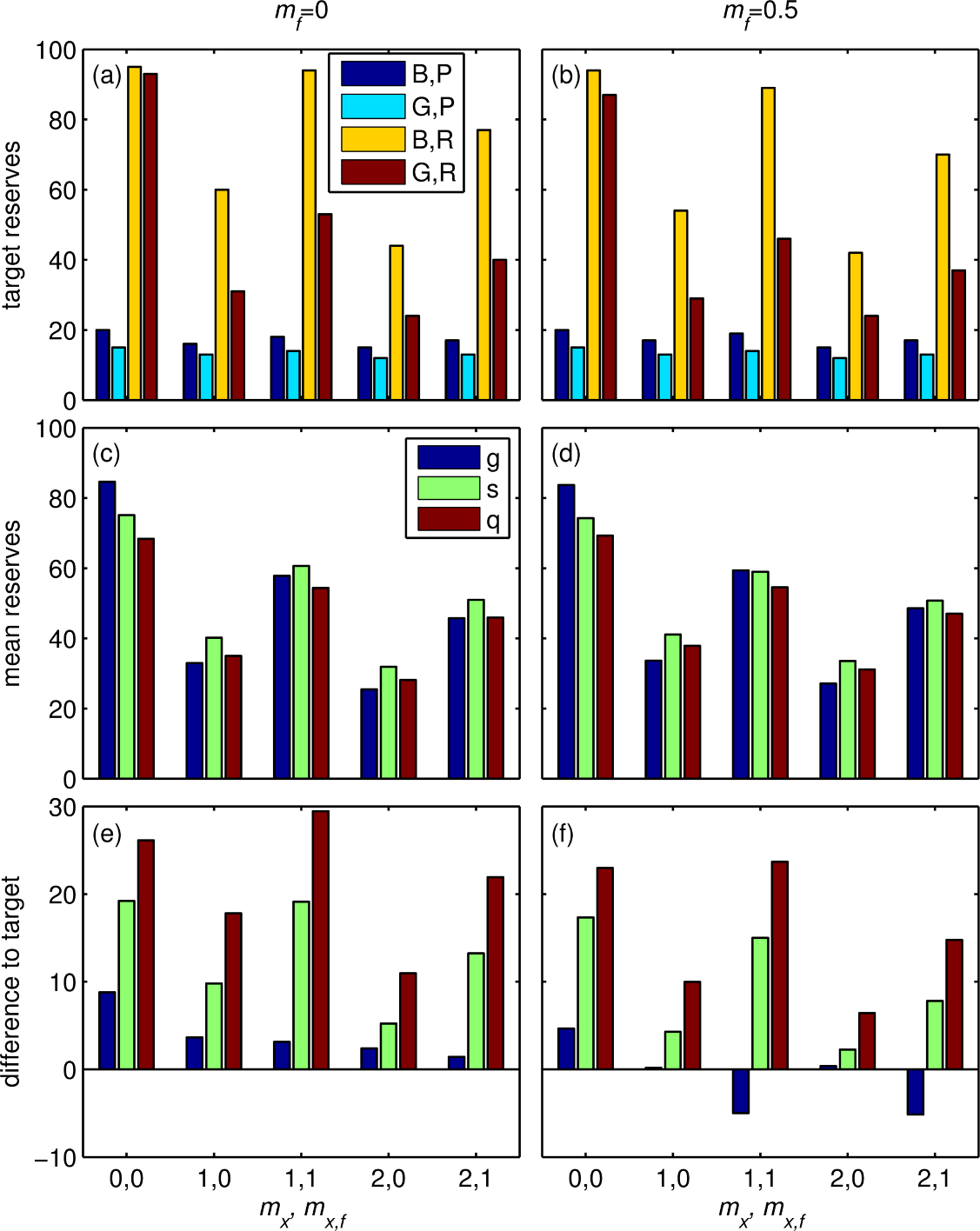

Supplement: Supplementary Data [file supp_eow031_Supp_data.doc]
